# Audio Deepfake Detection: What Has Been Achieved and What Lies Ahead

**DOI:** 10.3390/s25071989

**Published:** 2025-03-22

**Authors:** Bowen Zhang, Hui Cui, Van Nguyen, Monica Whitty

**Affiliations:** Department of Software Systems and Cybersecurity, Faculty of IT, Monash University, Melbourne, VIC 3800, Australia; bowen.zhang1@monash.edu (B.Z.); van.nguyen1@monash.edu (V.N.); monica.whitty@monash.edu (M.W.)

**Keywords:** audio deepfake detection, text to speech (TTS), voice conversion (VC), survey

## Abstract

Advancements in audio synthesis and manipulation technologies have reshaped applications such as personalised virtual assistants, voice cloning for creative content, and language learning tools. However, the misuse of these technologies to create audio deepfakes has raised serious concerns about security, privacy, and trust. Studies reveal that human judgement of deepfake audio is not always reliable, highlighting the urgent need for robust detection technologies to mitigate these risks. This paper provides a comprehensive survey of recent advancements in audio deepfake detection, with a focus on cutting-edge developments in the past few years. It begins by exploring the foundational methods of audio deepfake generation, including text-to-speech (TTS) and voice conversion (VC), followed by a review of datasets driving progress in the field. The survey then delves into detection approaches, covering frontend feature extraction, backend classification models, and end-to-end systems. Additionally, emerging topics such as privacy-preserving detection, explainability, and fairness are discussed. Finally, this paper identifies key challenges and outlines future directions for developing robust and scalable audio deepfake detection systems.

## 1. Introduction

Deepfake technology represents an intersection of deep learning and forgery techniques. The term “deepfake” gained widespread attention in 2017 when a Reddit user under the same moniker shared a pornographic video featuring celebrity faces generated using artificial intelligence (AI), which quickly went viral within the Reddit community [1,2,3]. While originally associated with visual media, the concept has since expanded to include audio deepfakes, which utilise advanced methods such as AI, machine learning, and neural networks to manipulate or synthesise deceptive audio content. These technologies were initially developed for positive applications such as education and entertainment [4], but their ability to generate highly realistic and convincing audio has led to misuse, such as scams [5]. Specifically designed to exploit vulnerabilities in either the human auditory perception system or automatic speaker verification (ASV) systems, these audio deepfakes pose severe threats when used for malicious or fraudulent purposes. The rapid expansion of interconnected devices in the Internet of Things (IoT) ecosystem has further amplified the risks associated with audio deepfake attacks. Modern sensor networks, particularly those deployed in security-sensitive applications such as smart surveillance [6,7,8], industrial monitoring [9,10], and biometric authentication [11,12,13,14,15], rely heavily on accurate voice data for identity verification and anomaly detection. However, the presence of deepfake-generated audio introduces new attack vectors, potentially compromising the integrity of these sensor-driven systems. Ensuring the robustness of sensor networks against adversarial deepfake manipulation is therefore critical to maintaining their reliability and security [16].

The emergence of audio deepfake technology is transforming the application of speech technologies. While its widespread use has brought convenience, it has also raised unprecedented security and ethical challenges. Using text-to-speech (TTS) or voice conversion (VC) techniques, deepfake technology can mimic specific individuals’ voices, such as political figures [17,18], enabling the spread of misinformation and even interfering with elections [19,20]. A report [21] indicates that TTS technology has been used to generate fake audio of U.S. president Joe Biden, urging voters to skip the 2024 New Hampshire primary election. Similar cases include the use of fake audio in cybercrime, where scammers impersonate individuals or company CEOs to deceive financial officers into transferring money [22]. These incidents highlight the potential risks of audio deepfakes, particularly in political, economic, and social domains. In addition, audio deepfake threats extend to real-time sensor network applications. For example, edge computing in wireless sensor networks has increasingly incorporated voice-activated interfaces to enable efficient human–device interaction. However, adversarial deepfake audio could deceive these interfaces, leading to unauthorised access or disrupted operations [23,24]. Moreover, embedded networked sensors in smart environments, such as those used in industrial IoT (IIoT) and healthcare applications, must be resilient against deepfake audio to ensure accurate anomaly detection and decision-making processes [25]. Despite the rapid advancements in deepfake technology, detection remains a severe challenge. A study [26] found that humans could correctly identify deepfake audio with an accuracy of only 73%. Another large-scale user study [27] involving over 1200 participants showed that while humans can rely on intuition and linguistic features for classification, their overall performance remains suboptimal in certain scenarios, particularly in complex environments. Furthermore, while existing machine learning models have achieved considerable progress, they have not consistently outperformed human intuition [27].

Unlike deepfake detection for images and videos, which has been intensively studied [28,29,30,31], audio deepfake detection has been overlooked in most of the existing surveys until recently [32]. Several survey papers have systematically summarised advancements in this field. For example, Almutairi and Elgibreen [33] categorised and quantitatively compared detection methods proposed between 2018 and 2021, and Khanjani et al. [32] analysed over 150 studies from 2016 to 2021, comprehensively summarising the frameworks of audio deepfake generation and detection while proposing potential defence strategies. In addition, Patel et al. [34] conducted a broad review of deepfake generation and detection technologies in 2023, analysing current challenges and research directions through case studies, and Yi et al. [35] provided a detailed classification of feature extraction methods, classification algorithms, and datasets, offering insights into key challenges and potential directions by comparing the generalisation capabilities of various detection methods. Despite these efforts, prior survey papers focus primarily on summarising audio deepfake detection techniques and datasets, often offering limited exploration of broader research directions beyond detection. Furthermore, these works often fall short in capturing the latest advancements in this rapidly evolving field. In this paper, in addition to systematically summarising the latest advancements in audio deepfake detection, we provide an in-depth review of advanced research directions, such as privacy protection and fairness. Furthermore, this survey analyses the limitations of current techniques and proposes targeted future research directions, aiming to serve as a comprehensive and valuable resource for researchers and practitioners in the field. A detailed comparison of these survey papers with ours is presented in Table 1.

This paper is organised as follows: Section 2 provides an overview of audio deepfake generation techniques and highlights their applications and potential risks. Section 3 reviews publicly available datasets used for training and evaluating detection models, analysing their characteristics and real-world relevance. Section 4 and Section 5 focus on evaluation metrics and methodologies of audio deepfake detection models. Section 6 explores advanced topics including privacy-preserving detection, fairness, adaptability, explainability, anti-spoofing strategies, and robustness against compression. Section 7 summarises key challenges in the field and outlines potential future research directions to guide further development in audio deepfake detection, and finally, Section 8 concludes this paper.

## 2. Overview of Audio Deepfake Methodologies

In the existing literature [4,32,33,34,35,36,37,38], audio deepfake technology is typically classified into two primary categories: speech synthesis and voice conversion. Speech synthesis focuses on generating entirely new speech from textual input, while voice conversion involves modifying the vocal characteristics of a source speaker to mimic a target speaker’s voice. Notably, voice conversion encompasses techniques such as imitation [33] and impersonation [32], especially when augmented using AI. Additionally, audio deepfake technologies can be further classified based on application scenarios into three subcategories: emotion fake, which alters the emotional tone of speech; scene fake, which fabricates or modifies audio to simulate specific environmental contexts; and partially fake, where only certain segments of an audio clip are manipulated while others remain genuine [35]. Each of these categories presents unique technical challenges and ethical concerns, underscoring the complexity and diversity of the field.

**Figure 1:**
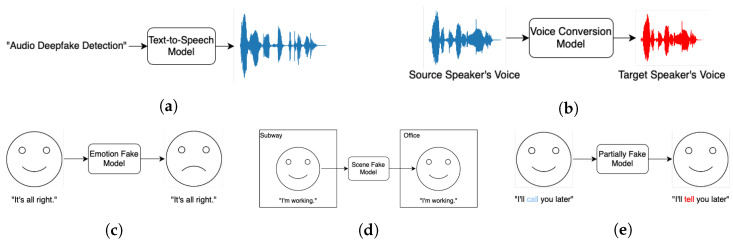
Overview of Different audio deepfake technology: (**a**) text-to-speech (TTS); (**b**) voice conversion (VC); (**c**) emotion fake; (**d**) scene fake; (**e**) partially fake.

This paper will primarily focus on speech synthesis and voice conversion, as they represent the most advanced and widely deployed approaches in the field. Replay attacks [32,33,36] and adversarial attacks [36] are commonly discussed in the literature but are not the focus of this paper as they do not always rely on AI [32]. A conceptual overview of these techniques and their workflows is presented in Figure 1, and a comparison between speech synthesis and voice conversion is shown in Table 2.

### 2.1. Speech Synthesis

Speech synthesis, also known as text-to-speech (TTS) conversion, is a technology that converts specific textual input into speech, closely resembling the voice of the target speaker. Traditional speech synthesis methods, including parametric synthesis and concatenative synthesis, have historically been capable of generating intelligible and fluent speech. However, the output produced this way often lacked human-like naturalness, resulting in mechanical or “robotic” sound [39]. The advent of deep learning marked a transformative shift in the field. In 2016, Google introduced WaveNet [40], a fully probabilistic, autoregressive waveform generation model. WaveNet demonstrated a performance that far surpassed traditional synthesis methods by producing speech with unprecedented naturalness and expressiveness. This breakthrough established a new benchmark for speech synthesis, inspiring the development of end-to-end (E2E) models such as DeepVoice 3 [41], Tacotron 2 [42], and FastSpeech 2 [43]. These models significantly reduced reliance on linguistic knowledge, thereby effectively lowering the technical barriers to high-quality speech synthesis. In recent years, research [44,45,46,47,48,49,50,51] has continued to refine these technologies, enabling synthesised speech to sound increasingly natural while also improving synthesis speed. This progress continues to push technological boundaries and meanwhile enables real-time applications, addressing a critical challenge in the deployment of TTS systems. The rapid evolution of TTS presents not only opportunities but also challenges for deepfake detection systems, which must now contend with increasingly sophisticated synthetic audio. This dual-edged nature of speech synthesis underscores its pivotal role in the broader landscape of audio deepfakes.

### 2.2. Voice Conversion

Voice conversion (VC), also known as speech-to-speech conversion, is a technology that modifies the vocal characteristics of a source speaker to emulate those of a target speaker while preserving the original linguistic content [35]. This process seamlessly integrates the unique vocal identity of the target speaker, making it a valuable tool in various applications, including personalised voice assistants [52], voice dubbing for cultural localisation in the entertainment industry [53], communication aids for individuals with speech impairments [54], and speaker de-identification for privacy and security purposes [55]. Conventional VC systems relied on a modular architecture comprising three components: speech analysis, conversion modelling, and speech reconstruction. The analysis phase extracted acoustic features from the source speech. These features were then transformed into the target speaker’s characteristics through a conversion model, and the modified speech was finally reconstructed using a vocoder [56]. While effective, these systems faced limitations, including dependency on extensive linguistic knowledge and suboptimal naturalness in the output. Benefiting from advancements in neural vocoders, modern VC systems have redefined their architecture. Since a neural vocoder itself is trainable, it enables joint training with conversion models [57] and, in some cases, speech analysis modules [58], achieving an E2E structure. Recent research [59,60,61,62,63] in VC technologies has addressed long-standing challenges such as data scarcity, cross-lingual conversion, and real-time processing. Despite its technical advancements, voice conversion raises critical ethical and societal concerns. The ability to create high-fidelity voice clones poses significant risks of identity fraud and misinformation, particularly in scenarios involving deepfakes. For instance, an adversary could exploit VC technologies to impersonate individuals, thereby undermining trust in voice-based communication. Additionally, privacy risks are also notable, as ASV systems could be exploited to bypass biometric authentication. These challenges necessitate robust safeguards and the development of countermeasures to detect and mitigate misuse.

## 3. Dataset

Datasets are fundamental to progress in audio deepfake detection, serving as the basis for training, validating, and benchmarking detection algorithms. High-quality datasets empower researchers to assess the performance and generalisability of their methods across a spectrum of forgery techniques, linguistic variations, and recording conditions [64]. As deepfake technologies grow increasingly sophisticated, annotated datasets become indispensable for rigorous testing against realistic and complex manipulations. This section provides an overview both of representative and of some of the recently proposed datasets in the field of audio deepfake detection, highlighting their contributions to advancing detection capabilities. The baseline performance on the competition dataset is presented in Table 3, and the description of additional datasets is provided in Table 4.

### 3.1. ASVspoof Challenge

Automatic Speaker Verification Spoofing and Countermeasures Challenge (ASVspoof www.asvspoof.org (accessed on 17 December 2024)) stands as the largest and most comprehensive international competition in the field of spoofed speech detection. Its primary objective is to enhance the resilience of ASV systems against spoofing attacks. Since its beginning in 2015, ASVspoof has conducted multiple editions—in 2015, 2017, 2019, 2021, and 2024—each contributing significantly to the advancement of spoofed speech detection technologies.

Previous research on spoofing attacks and countermeasures has relied on full knowledge of specific ASV systems for vulnerability assessments. Similarly, countermeasures were designed with prior knowledge of the spoofing attacks they aimed to detect, including the type of attack and even the specific algorithms used [65]. This approach, however, is inconsistent with real-world scenarios, where neither the exact nature of the attack nor its underlying algorithms are known in advance. To address these limitations, ASVspoof 2015 [66] was introduced. The ASVspoof 2015 dataset includes samples generated through speech synthesis and voice conversion, with genuine speech recordings from 106 speakers. However, due to the dataset’s age, it is rarely used in contemporary research.

ASVspoof 2017 [67,68] focuses primarily on replay attacks under various conditions. The dataset was developed based on the RedDots corpus [69] and its replayed version [70], comprising genuine audio recordings from 42 speakers and replayed audio from 177 replay sessions, covering 61 distinct replay configurations. This design enables a comprehensive evaluation of the robustness of ASV systems against replay attacks across varying acoustic environments and playback device characteristics.

ASVspoof 2019 [71,72] introduced for the first time a distinction between two types of spoofing attacks: Logical Access (LA) and Physical Access (PA). LA involves directly injecting spoofed audio into the ASV system, utilising techniques such as TTS and VC. In contrast, PA deceives the ASV system by replaying spoofed audio through devices like loudspeakers. This dataset was constructed using the VCTK corpus [73], featuring recordings from 107 speakers. It incorporated state-of-the-art neural acoustic and waveform models at the time to generate spoofed audio and employed more controlled replay attack methods.

ASVspoof 2021 [74,75] built upon the ASVspoof 2019 framework by introducing, for the first time, a Speech Deepfake (DF) detection task aimed at identifying spoofed speech outside ASV scenarios. With no new training or development partitions provided, the challenge included three new progress and evaluation partitions for each task. The LA task incorporated the effects of telephony encoding and transmission, simulating more realistic communication scenarios. The PA task introduced reverberation and additive noise, challenging participants to address attacks under varying physical conditions. The DF task utilised additional undisclosed corpora and various lossy codecs to better simulate real-world media storage and transmission.

The ASVspoof 5 [76] dataset represents the largest and most complex design in the history of the ASVspoof challenges. Taking the Multilingual LibriSpeech (MLS) [77] English dataset as its source data, it introduced crowdsourced data for the first time to simulate a diverse range of real-world scenarios, along with adversarial attacks (ADs), to evaluate system robustness under extreme conditions. Unlike previous editions, ASVspoof 5 no longer focuses on replay attacks. Instead, the challenge is divided into two tracks: countermeasure (CM), which addresses standalone spoofing and deepfake speech detection, and spoofing-robust ASV (SASV), which focuses on adversarial attacks within the context of ASV systems. The dataset encompasses a wide variety of spoofing techniques, including state-of-the-art TTS and VC. It also simulates various acoustic environments and device conditions, offering a comprehensive evaluation framework for modern spoofing detection systems.

### 3.2. ADD Challenge

Audio Deep Synthesis Detection Challenge (ADD http://addchallenge.cn (accessed on 17 December 2024)) is another international competition in the field of spoofed speech detection, with a stronger focus on real-life audio deepfakes compared to the studio-quality data emphasised by ASVspoof 2021. Since its inception in 2022, the ADD Challenge has successfully held two editions, fostering advancements in detecting audio deepfakes under more realistic conditions.

ADD 2022 [78] contained three tracks: Low Quality (LF), Partially Fake (PF), and Audio Fake Game (FG). LF focused on distinguishing audio containing real-world noise, background music, and other effects. PF aimed to detect partially forged audio, where only segments of a recording had been manipulated. FG consisted of two adversarial tasks: audio generation (FG-G) and audio detection (FG-D). In FG-G, participants were tasked with generating audio capable of deceiving FG-D systems, while FG-D participants were required to detect both the official evaluation set and submissions from FG-G participants. Some of the dataset audio was sourced from the AISHELL corpus [79,80,81].

ADD 2023 [82] retained the FG track while further refining the detection of audio deepfakes. It introduced two new tasks: Manipulation Region Location (RL), which focused on identifying the boundaries of partially forged audio, and Deepfake Algorithm Recognition (AR), aimed at recognising the specific algorithms used to generate deepfake audio. In addition to the datasets utilised in ADD 2022, the THCHS-30 corpus [83] was also incorporated, enhancing the diversity and robustness of the challenge data.

### 3.3. Additional Datasets

The consecutive organisation of the ASVspoof and ADD challenges has significantly propelled the development of audio deepfake detection technologies. These competitions have also inspired the creation of more diverse, noncompetition datasets, addressing limitations such as the monolingual focus often seen in competition datasets. Over time, researchers have developed a series of datasets to enrich the field with diverse and high-quality resources, filling the gaps in existing datasets.

**Table 4:** Overview of additional datasets and their characteristics.

Name	Language	Year	Fake Types	Key Improvement
FoR [84]	English	2019	TTS	Expands data volume, covers latest TTS technology
WaveFake [85]	English, Japanese	2021	TTS	Encompasses state-of-the-art generative models
HAD [86]	Chinese	2021	Partially fake	Focuses on detecting partially fake audio
ITW [64]	English	2022	TTS	Provides audio recorded in the wild
LibriSeVoc [87]	English	2023	Self-vocoding	Includes neural vocoder artefacts in audio samples
SceneFake [88]	English	2024	Scene manipulation	Detects scene forgery in audio
EmoFake [89]	English, Chinese	2024	EVC	Focuses on emotional change in speech
CVoiceFake [90]	English, Chinese, German, French, Italian	2024	TTS	Multilingual, provides ground-truth transcriptions
MLAAD [91]	38 languages	2024	TTS	Multilingual, offers support for global applicability

In 2019, to address the limited coverage of the latest TTS technologies and the insufficient data volume for training complex models, Reimao and Tzerpos introduced the Fake or Real (FoR) [84] **dataset**. FoR is a high-quality, diverse dataset focused on synthetic speech detection, comprising four versions that include various recording conditions, balanced genders, and different noise levels, meeting multiple research needs in audio deepfake detection. In 2021, Frank and Schönherr released the WaveFake [85] dataset to further advance the study of audio deepfake detection. This dataset includes audio samples generated from six different neural architectures, encompassing state-of-the-art generative models. WaveFake provides significant diversity, laying a solid foundation for studying the generalisation and robustness of audio deepfake detection methods.

Existing datasets primarily focus on fully spoofed audio, often overlooking scenarios involving partially fake audio, where fake segments are embedded with genuine speech. To address this challenge, Yi et al. introduced the Half-Truth Audio Detection (HAD) [86] **dataset**. HAD not only focuses on detecting partially fake audio but also provides location information for fake segments, enabling more subtle evaluations. In 2022, Müller et al. [64] pointed out that most audio deepfake detection models are trained and evaluated on the ASVspoof dataset, which includes limited spoofing algorithms and is based on the VCTK corpus recorded in controlled studio environments. These constraints hinder the models’ ability to generalise to real-world deepfake audio. To address this issue, the authors developed the In-the-Wild dataset (ITW), consisting of genuine audio from public speeches, interviews, and natural recording scenarios of 58 politicians and celebrities, alongside deepfake audio generated using diverse synthesis techniques.

In 2023, Sun et al. [87] proposed a method for detecting spoofed audio by identifying traces of neural vocoder artefacts. Based on this approach, they introduced the LibriSeVoc dataset, which includes audio samples generated using six neural vocoders and genuine speech, providing a valuable benchmark for detecting vocoder-generated fake audio. In 2024, Yi et al. [88] introduced the SceneFake dataset, focusing on detecting scene forgery in audio. SceneFake includes samples where only the acoustic scene is altered while keeping the speech content unchanged, filling a critical gap in existing audio deepfake detection datasets. Current datasets often neglect the problem of emotional change in speech. To address this, Zhao et al. [89] developed the EmoFake dataset based on the Emotional Speech Database (ESD). EmoFake simulates scenarios of emotional transformation and uses seven advanced emotional voice conversion (EVC) models to generate fake audio samples.

Language coverage in existing datasets remains limited, primarily favouring English and Chinese, which restricts the generalisation of detection models in multilingual scenarios. To address this, Li et al. [90] created the CVoiceFake dataset, covering five languages and various deepfake techniques. CVoiceFake also provides ground-truth transcriptions to support the evaluation of content preservation in fake audio. Similarly, Müller et al. [91] developed the Multi-Language Audio Anti-Spoofing Dataset (MLAAD), a large-scale multilingual dataset covering 38 languages and 82 spoofing models, offering robust support for the global applicability of audio deepfake detection systems.

## 4. Evaluation Metrics

As a binary classification task, audio deepfake detection often encounters the problem of imbalanced data distribution, where the proportion of genuine and fake samples is uneven. In such scenarios, simple metrics like accuracy tend to be biased towards the majority class, failing to provide a comprehensive evaluation of system performance. Moreover, accuracy cannot effectively assess the overall performance of a detection system when integrated into an ASV framework. To address these limitations, this field typically employs metrics such as Equal Error Rate (EER) and Tandem Decision Cost Function (t-DCF), which offer a more balanced and holistic evaluation of both the standalone detection system and its contribution to the performance of an integrated ASV system.

### 4.1. Equal Error Rate (EER)

EER is a widely used metric for evaluating ASV and other biometric systems in binary classification tasks. It represents the error rate at the threshold where the False Acceptance Rate (FAR) equals the False Rejection Rate (FRR). In the context of detecting audio deepfakes, FAR is the probability of misclassifying an actual fake audio sample as genuine, while FRR is the probability of misclassifying an actual genuine audio sample as fake.

Given all the detection scores of a particular system, the FAR and FRR at a specific threshold θ can be defined as [66]

$$FAR\left(\theta \right)=\frac{\#\{fake\;trials\;with\;score>\theta \}}{\#\{total\;fake\;trials\}},$$

$$FRR\left(\theta \right)=\frac{\#\{genuine\;trials\;with\;score\leq \theta \}}{\#\{total\;genuine\;trials\}},$$

where FAR(θ) and FRR(θ) are monotonically decreasing and increasing functions of θ, respectively, and the EER is where the two curves intersect. One may also see the EER as the crossover error rate [92] and the FAR and FRR as the false alarm rate and miss rate [66] in other studies.

In achieving a low EER, the goal is to minimise both the FAR and FRR simultaneously, indicating that the system can maintain a balance between sensitivity and specificity while achieving a low overall error rate. The EER is typically expressed as a percentage, with lower values indicating better system performance.

### 4.2. Tandem Decision Cost Function (t-DCF)

The EER primarily reflects the standalone performance of a spoof detection system without fully considering its integration and real-world application within an ASV system. In practical scenarios, spoof detection modules typically operate as auxiliary components within ASV frameworks, working collaboratively to form a unified system. However, the independent performance of a spoof detection system does not necessarily align with the overall effectiveness of the integrated ASV system, leading to challenges in evaluating their combined performance. To address this limitation, Kinnunen et al. [93] proposed the Tandem Decision Cost Function (t-DCF), a comprehensive evaluation metric that assesses both the standalone performance of spoof detection systems and their impact on the overall ASV system. In real-world applications, ASV systems typically process three types of inputs: bona fide target, bona fide nontarget, and spoofed target. The t-DCF metric quantifies the contribution of the spoof detection module to the overall ASV system’s performance, thereby offering a more holistic assessment. A simplified t-DCF calculation is given as [36]

$$min\;t-\mathrm{DCF}=\underset{\theta }{min}\left\{FAR\left(\theta \right)+\beta \;FRR\left(\theta \right)\right\}$$

where β depends on factors such as the priority of spoof attacks, misclassification costs, and the performance of the ASV system. A lower t-DCF indicates better overall performance and generalisability. For detailed derivations and computation methods, readers are referred to [93].

The t-DCF was first introduced as the primary evaluation metric in ASVspoof 2019. In ASVspoof 5, it is used concurrently with the recently proposed t-EER [94] and a-DCF [95], reflecting ongoing improvements in assessing the effectiveness of integrated ASV and spoof detection systems.

## 5. Audio Deepfake Detection

**Figure 2:**
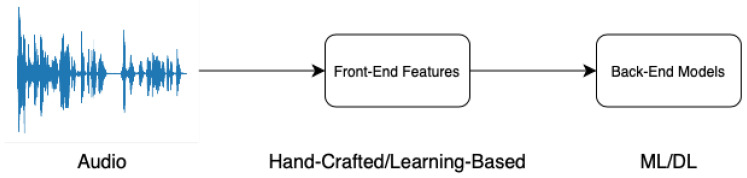
Overall workflow of audio deepfake detection. If the raw waveform is directly fed into the backend classification model, the model is considered an end-to-end approach.

Detecting audio deepfakes has become a critical area of research, as advancements in audio synthesis and manipulation technologies increasingly threaten security, privacy, and trust in digital media. Early detection efforts primarily focused on protecting ASV systems from spoofing attacks. However, with the rapid evolution of deepfake technologies, the use of fake audio to spread misinformation has emerged as a significant concern. In the pipeline of detection methods, a model is typically divided into two components: frontend feature extraction and backend classification. Figure 2 illustrates the overall workflow of audio deepfake detection. This section examines the core aspects of audio deepfake detection, including these two components, in detail.

### 5.1. Frontend Features

Feature extraction is a pivotal component in the pipeline of audio deepfake detection, as a significant body of research [64,96,97,98,99] has demonstrated that the performance of detection models largely depends on the choice of input features. Given the diverse techniques used to generate fake audio, selecting robust features that can capture subtle artefacts becomes a challenging yet crucial task. The input features for audio deepfake detection models can be generally categorised into two types: handcrafted features and learning-based features.

#### 5.1.1. Handcrafted Features

Handcrafted features rely on domain expertise in audio signal processing to manually design fixed feature representations derived from time, frequency, or cepstral domains. They have been widely used in traditional audio analysis tasks and remain relevant in audio deepfake detection due to their interpretability and relatively low computational requirements. In recent years, commonly used handcrafted features can be generally categorised into time–frequency domain features and cepstral domain features.

Time–frequency domain features, derived through Short-Time Fourier Transform (STFT) or other time–frequency analysis techniques, describe signal characteristics by simultaneously considering both time and frequency information. These features are particularly effective for analysing and processing nonstationary signals [100].

Log Power Spectrum (LPS) is derived from the STFT of an audio signal, followed by logarithmic transformation and power calculation. This feature effectively captures the time–frequency dynamics of audio signals while enhancing weak signals and compressing strong ones. This process results in a more intuitive and manageable spectral representation of audio data. Zhang et al. [101] employed LPS as a primary frontend feature and introduced a method involving per-band processing and multiband fusion. This approach significantly improved the robustness and generalisation ability of the detection system. Experimental results demonstrated that low-frequency features performed better in detecting known attack types. These findings underscore the strong potential of LPS, particularly when combined with multiband fusion techniques.

Linear Filter Bank (LFB), a simplified version of STFT, offers reduced computational complexity and lower risks of overfitting during training. Chen et al. [102] employed augmented LFB features to train a deep residual network (ResNet), resulting in a model with strong generalisation capabilities, underscoring the balance between computational efficiency and spectral representation provided by LFB. Building on the effectiveness of LFB, Tak et al. [103] used log-scale LFB with a ResNet for embedding extraction and employed a graph attention network (GAT) to model temporal and spectral relationships. This approach achieved SOTA performance in the ASVspoof 2019 LA dataset. In a comparative study, Wang and Yamagishi [98] evaluated several frontend features, including LFB. While LFB demonstrated strong performance in ResNet-based models, it was not the optimal choice for LCNN-based architectures.

Mel Spectrogram is a time–frequency domain feature representation based on the Mel frequency scale, which emulates the human auditory system’s frequency perception. By mapping the spectrum onto the Mel scale, it captures the frequency characteristics of a signal while reducing high-frequency resolution to focus on frequency ranges sensitive to human hearing. This property has made the Mel Spectrogram a mainstream feature in speech processing and audio analysis. Fathan et al. [104] explored the use of Mel Spectrograms combined with VGG16 and WaveletCNN architectures, significantly outperforming traditional cepstral features in detecting LA attacks. Similarly, Wani et al. [105] utilised an Attention-Based Cascaded Capsule Network (ABC-CapsNet) as a backend model, achieving exceptional results that surpassed other SOTA methods. These findings underscore the Mel Spectrogram’s ability to retain critical frequency and temporal patterns, enhancing its utility in audio deepfake detection.

Unlike STFT, Constant-Q Transform (CQT) provides higher frequency resolution in the low-frequency range and higher time resolution in the high-frequency range. This characteristic makes CQT well suited for analysing nonlinear frequency features in music and speech signals. Li et al. [106] also used CQT in their study, showing that CQT achieved a significantly lower EER compared to Spec, indicating superior generalisation to unseen attack types. Furthermore, CQT’s ability to capture fine-grained spectral details across a broader frequency range enhanced its robustness against diverse deepfake techniques. Traditional CQT features, however, neglect phase information, which is critical for identifying artefacts in synthetic speech. To overcome this limitation, Müller et al. [107] proposed Complex-Valued CQT Spectrograms (C-CQTs), which retain both magnitude and phase information. Using a complex-valued neural network (CVNN) classifier based on CNNs, this approach achieved SOTA performance on the ASVspoof2019 LA and IWA datasets, highlighting its robustness and suitability for detecting synthetic speech artefacts.

Log Power Magnitude Spectrogram (Spec) is a logarithmic representation of the spectrogram obtained through STFT. By squaring the magnitude spectrum and applying a logarithmic transformation, Spec mimics the nonlinear perception of human hearing, enhancing low-frequency signals while compressing high-frequency components. In the study conducted by Li et al. [106], Spec, along with several other acoustic features, was evaluated using the SE-Res2Net50 architecture. Experimental results indicated that Spec struggled with generalisation when facing synthetic speech. This limitation was attributed to the absence of phase information in Spec features, as many spoofing cues for LA attacks are embedded in the phase domain. To address this issue, Ling et al. [108] proposed an attention-based convolutional neural network (CNN) to enhance Spec representations. The attention mechanism ensured that the system emphasised critical regions of the spectrogram, leading to improved deepfake detection performance. The proposed model outperformed all single systems in the ASVspoof 2019 LA dataset, highlighting the effectiveness of Spec when combined with attention-based enhancements for capturing subtle spectral anomalies.

Cepstral Domain Features extract key spectral characteristics of audio signals by analysing the logarithmic energy distribution of their spectra. Typically, the computation involves applying a logarithmic transformation to the spectrum, followed by an inverse Fourier transform or discrete cosine transform (DCT). This process effectively separates spectral envelope information from fine spectral details, making cepstral features particularly valuable in speech signal processing [109]. These features not only capture hidden artefacts in deeply forged signals but also offer a compact and meaningful representation.

Mel-Frequency Cepstral Coefficients (MFCC) are a cepstral feature based on the Mel frequency scale. By transforming spectral information into a compact cepstral representation, MFCC effectively capture the core spectral characteristics of speech. Zhang et al. [110] utilised MFCC as a frontend feature in their TE-ResNet framework, achieving near-optimal detection performance. The robustness of MFCC-derived spectral features contributed to the model’s stable performance, making it competitive with SOTA methods. Hamza et al. [111] further investigated MFCC in various machine learning and deep learning frameworks, demonstrating its effectiveness in distinguishing genuine and fake audio. Their experiments revealed that while traditional machine learning models performed well on clean datasets, deep learning models exhibited superior robustness in more complex data scenarios. Firc et al. [112] expanded the application of MFCC by introducing the MFCC-Cepstrogram, a visualised cepstral feature representation used as input to Temporal Convolutional Networks (TCNs). This approach examined the impact of different spectrogram features on audio deepfake detection. Experimental results on the ASVspoof 2019 LA and FoR datasets indicated that MFCC-Cepstrogram achieved exceptional performance and exhibited better stability on unseen data compared to other spectral features. This highlights MFCC-Cepstrogram’s computational efficiency and adaptability, especially in resource-constrained scenarios. Moreover, the study demonstrated that enhancing MFCC features through visualisation can further improve detection performance.

Similar to MFCC, Linear Frequency Cepstral Coefficients (LFCC) utilise cepstral analysis but replace the Mel frequency scale with a linear frequency scale, enabling them to retain more detailed information in the high-frequency regions. This makes LFCC particularly suitable for detecting spectral distortions in deepfake audio. Zhang et al. [113] leveraged LFCC within a one-class learning framework combined with a ResNet for feature processing. Their experiments significantly outperformed traditional LFCC-GMM approaches, revealing LFCC’s superior ability to capture high-frequency artefacts. Additionally, the one-class learning framework demonstrated LFCC’s potential in handling unknown attack types. Luo et al. [114] adopted LFCC as a frontend feature and proposed a backend framework based on capsule networks. The combination of capsule networks and LFCC significantly improved the generalisation ability to unknown attacks, highlighting its strong potential in deepfake audio detection. Furthermore, Wang et al. [115] introduced Multiscale Permutation Entropy (MPE) to complement LFCC by capturing the dynamic structural information lost during extraction. By directly integrating MPE with LFCC, they developed a more expressive joint feature representation. This study highlights the enduring value of handcrafted features when integrated with novel methodologies, particularly in resource-constrained environments where large-scale pretraining is infeasible.

Constant-Q Cepstral Coefficients (CQCC), derived from CQT, combine the multiresolution characteristics of CQT with the compactness of cepstral features, making them highly effective for capturing the time–frequency characteristics of audio signals. Zhang et al. [110] explored CQCC alongside MFCC, revealing their significant advantages in multiresolution analysis. CQCC demonstrated superior performance in capturing complex spectral structures and detecting deepfake artefacts, making them a preferred choice for scenarios requiring detailed spectral representation. Their ability to efficiently encode time–frequency information underscores their robustness in audio deepfake detection tasks.

#### 5.1.2. Learning-Based Features

While handcrafted features, extracted manually using digital signal processing algorithms, have demonstrated significant utility in audio deepfake detection, their limitations are also evident. These features often fail to capture nuanced and potentially useful features that are critical for distinguishing genuine audio from deepfakes. Moreover, their generalisation ability is typically constrained, particularly when dealing with out-of-domain scenarios or novel attack types [116]. In contrast, learning-based features have emerged as a powerful alternative, enabling models to automatically learn discriminative representations directly from raw audio or spectrograms. By leveraging large-scale datasets, these methods can uncover complex patterns and subtle artefacts that are difficult to design manually. Consequently, deep learning approaches have increasingly become the mainstream solution in audio deepfake detection, offering superior performance and robustness against diverse and evolving deepfake technologies [117].

In the field of audio deepfake detection, supervised learning methods such as SincNet and RawNet2 have demonstrated the ability to directly learn task-specific features from raw waveforms. Compared to traditional CNNs with fully parameterised convolutions, SincNet [118] employs filters based on parameterised sinc functions, offering a more compact and efficient approach to bandpass filtering. By optimising only the low and high cutoff frequencies of the filters, SincNet significantly reduces model complexity while improving performance and convergence speed. For instance, Li et al. [119] combined SincNet-learned features with the Mel-Spectrogram to provide high-dimensional embeddings for subsequent classifiers, thereby enhancing the model’s robustness against diverse deepfake audio scenarios. RawNet [120], an E2E network designed for text-independent speaker verification, adopts CNNs in its first layer to extract temporal and spectral features from raw waveforms. Building upon this foundation, Jung et al. further introduced RawNet2 [121], which replaces CNN with SincNet to better capture aggregated frequency responses. Experimental results demonstrate that RawNet2 outperforms the original RawNet across multiple benchmark tests, validating the effectiveness of SincNet in enhancing frequency domain modelling. Further extending this utility, Ranjan et al. [122] employed RawNet2 as a backbone network to extract temporal and spectral features for multitask learning frameworks. Their findings highlight RawNet2’s robust feature extraction capabilities, which provide a solid foundation for improving the performance of audio deepfake detection.

Traditional supervised learning methods rely heavily on large-scale labelled data, which are both expensive and challenging to obtain for diverse scenarios. In contrast, self-supervised learning (SSL) adopts large volumes of unlabelled data for pretraining, learning deep representations through contextual information, thereby significantly reducing dependence on labelled datasets. In audio deepfake detection, self-supervised models demonstrate the capability to capture subtle artefacts and anomalies present in deepfake audio.

Wav2Vec 2.0 (W2V2) [123] is a Transformer-based self-supervised learning framework for speech processing. By directly modelling raw audio waveforms, W2V2 generates high-quality contextual representations similar to BERT, making it widely applicable to downstream speech tasks. An extension of the W2V2 framework, Cross-Lingual Speech Representations (XLSR), further enhances speech processing in multilingual environments through cross-lingual pretraining. Depending on the number of languages used for training, XLSR supports 53 languages (XLSR [124]) and 128 languages (XLS-R [125]). The study of Martín-Doñas et al. [126] explored both XLSR and XLS-R as feature extractors for audio deepfake detection. By integrating hidden representations from different Transformer layers, they achieved remarkable performance on the ASVspoof 2021 and ADD 2022 datasets, highlighting the capability of SSL-based features.

Similarly, Saha et al. [127] fed embeddings extracted from multiple Transformer layers of W2V2 Base directly into classical machine learning models, such as the Support Vector Machine (SVM) and Logistic Regression (LR). Experimental results showed that their method achieved competitive performance on the ASVspoof 2019 LA dataset compared to SOTA deep learning models while significantly reducing computational costs. These lightweight models do not require GPU resources, underscoring the effectiveness of pretrained features in audio deepfake detection and providing a valuable contribution to the development of Green AI.

WavLM [128], building on the W2V2 framework, introduces enhancements beyond masked speech prediction by incorporating a masked speech de-noising task. This modification enables WavLM to extract universal speech representations that generalise well across various speech tasks. Guo et al. [129] proposed a multifusion attentive (MFA) classifier to extract and integrate outputs from different Transformer layers and time steps of WavLM. Their results highlighted the potential of SSL models in audio deepfake detection, particularly in challenging acoustic environments.

Zhu et al. [130] proposed the Modulation Transformation Block (MTB) to address the absence of long-term temporal dynamics in the universal speech representations extracted by self-supervised speech models. Experimental findings revealed that the integration of MTB significantly improved WavLM’s detection performance on the ASVspoof 2021 dataset, outperforming W2V2 in overall effectiveness. This demonstrates the critical role of combining self-supervised models with targeted enhancements to address specific deficiencies, further advancing the performance in audio deepfake detection.

Moreover, Zhu et al. [131] proposed a two-stage framework named Style–Linguistics Mismatch (SLIM), which relies solely on authentic speech samples to learn the dependency between style and linguistic content through self-supervised pretraining. During inference, SLIM combines pretrained style–linguistics features with acoustic features to classify speech. With a frozen feature encoder, it achieves superior performance on out-of-domain datasets and competitive results on in-domain datasets. Additionally, SLIM provides interpretability by quantifying the alignment between style and linguistic content, helping to explain how a sample is identified.

In addition, feature extraction methods based on time–frequency representations have also gained widespread attention, particularly models with VGG networks. VGG [132] **networks** are well-known for their deep stacked convolutional architecture and robust image feature extraction capabilities. In audio deepfake detection, VGG networks are widely deployed as feature extractors for time–frequency representations, effectively identifying subtle artefacts in fake audio by capturing both local patterns and global correlations within the spectral features. Fathan et al. [104] used VGG16 as a feature extractor to analyse spectrograms, focusing on capturing artefacts in the frequency and temporal dimensions. Their findings demonstrated the potential of VGG16 combined with Mel-Spectrograms for detecting fake audio, particularly under complex scenarios such as channel mismatch, where the approach showed remarkable performance. Similarly, Mcuba et al. [133] conducted a comparative study on the performance of VGG16 combined with various audio representations, including the MFCC, Mel-Spectrogram, Chromagram, and spectrogram. The results revealed that VGG16 excelled on MFCC while also performing reasonably well on other features, highlighting its versatility across diverse input representations. Wani et al. [105] further combined VGG18 with Mel-Spectrograms and integrated it with attention mechanisms and capsule networks to enhance the robustness and performance of detection. Their approach achieved unprecedented low EERs on the ASVspoof 2019 LA dataset.

### 5.2. Backend Model

In audio deepfake detection tasks, backend models include both machine learning and deep learning approaches. Machine learning models primarily rely on handcrafted feature inputs, offering high computational efficiency and strong interpretability. As a result, they were widely adopted in early research. While machine learning models demonstrate notable robustness, neural network-based deep learning models generally perform better [85]. With the development of deep learning techniques, these models have become the mainstream solution due to their powerful capability to learn complex feature representations.

#### 5.2.1. Machine Learning

The Gaussian Mixture Model (GMM) is a probabilistic model and an extension of a single Gaussian probability density function designed to represent a mixture of multiple Gaussian distributions. By treating data as a weighted combination of several Gaussian distributions, GMM can effectively capture complex data distributions. It has been widely used in audio deepfake detection, speaker verification, and other pattern recognition tasks. Although deep learning models have become the mainstream in recent years, GMM remains valuable in specific tasks due to its efficiency and interpretability, and it is used as a standard baseline model in the ASVspoof Challenge and ADD Challenge. Apart from the GMM, other machine learning models, such as the Support Vector Machine and Logistic Regression, were widely studied in earlier research [96,134,135,136]. With the advancement of feature extraction techniques, these models continue to play a significant role in current studies [137,138,139].

#### 5.2.2. Deep Learning

Deep learning models can be broadly categorised according to their architectures, including CNN-based models, capsule network-based models, and graph neural network (GNN)-based models.

Convolutional neural networks (CNNs) are widely applied in pattern recognition and classification tasks due to their powerful feature extraction capabilities and computational efficiency. The core of CNNs lies in their hierarchical structure, combining convolutional layers, pooling layers, and fully connected layers to progressively extract features from input data, transitioning from low-level to high-level representations. In the field of audio deepfake detection, CNNs are particularly adept at capturing localised artefacts within spectro-temporal decompositions, such as spectrograms [103]. Light CNN (LCNN) builds upon traditional CNN architectures by introducing the Max-Feature-Map (MFM) activation function and smaller convolutional kernels, significantly reducing the number of parameters while maintaining a balance between utility and performance. LCNN has been extensively used as a baseline model in challenges like ASVspoof and ADD. For instance, Wang et al. [115] employed LCNN as a classifier to process fused LFCC and MPE feature vectors for deepfake audio detection. Their results demonstrated that LCNN effectively captures temporal and frequency patterns with fused features, significantly enhancing detection accuracy and robustness. ResNet, with its innovative Residual Learning Framework, addresses challenges such as gradient vanishing and degradation issues in deep networks. Initially achieving significant breakthroughs in image classification tasks on ImageNet, ResNet has also proven effective in audio applications. Zhang et al. [113] utilised a ResNet-18 architecture, replacing the standard global average pooling with attentive temporal pooling to better focus on time-dimensional features critical for deepfake detection. Combined with the newly proposed One-Class (OC) softmax loss function, this model further improved the differentiation between real and fake audio. Experimental results showed outstanding performance on unseen attacks, underscoring its strong generalisation capabilities. Another study [110] incorporated Transformer Encoders (TEs) to extract contextual representations of acoustic features, which were then fed into ResNet. The approach demonstrated remarkable performance across multiple datasets, outperforming CNNs and LCNN in terms of both accuracy and generalisation. Squeeze-and-Excitation Networks (SENets) are architectural units designed to enhance the representational capacity of neural networks. SENets capture global responses of channel features through the squeeze operation and generate channel-wise weights via the excitation operation, recalibrating each channel’s contribution dynamically. These modules can be seamlessly integrated into existing CNN architectures with minimal computational overhead. In Wang et al.’s study [115], SENet outperformed LCNN on the ASVspoof2019 and ITW datasets, and its combination with MPE exhibited strong generalisation capabilities.

Although traditional CNNs are capable of recognising images without precise spatial information, they struggle to effectively model the intricate spatial relationships between hierarchical components. Capsule networks (CapsNets) [140], on the other hand, address this issue by using dynamic routing algorithms to infer spatial information and other parameters, enabling more flexible and detailed object modelling. Luo et al. [114] refined the original dynamic routing algorithm to focus more effectively on deepfake artefacts in audio. In their study, CapsNets output feature vectors that not only capture the fake characteristics of audio but also provide classification probabilities. By incorporating random noise and an optimised softmax function, this approach significantly enhanced feature extraction accuracy and discriminative ability. Experimental results showed that this model outperformed baseline methods such as the GMM and LCNN on the ASVspoof 2019 LA task, offering a novel direction for audio deepfake detection. Additionally, Wani et al. [105] proposed a novel architecture called Attention-Based Cascaded CapsNet (ABC-CapsNet). This model utilised a multilayer processing framework to iteratively abstract structural information from audio data while detecting subtle artefacts in fake audio with the unique ability of CapsNet. Experimental results demonstrated that ABC-CapsNet achieved an extremely low EER on the ASVspoof 2019 dataset, further validating the exceptional performance and potential of CapsNets in addressing complex audio deepfake detection challenges.

With the remarkable success of self-attention-based Transformer models in natural language processing (NLP), the introduction of the Vision Transformer (ViT) [141] has further demonstrated the versatility of Transformers in computer vision tasks. Unlike traditional CNNs, the ViT eliminates the need for convolution operations by dividing an image into small patches and taking each patch as a token, which is then processed by a Transformer model. This approach overcomes the CNN’s limitations in local receptive fields by leveraging global attention to capture long-range dependencies, enabling the ViT to outperform CNNs on large-scale datasets. In the audio domain, one-dimensional audio signals can be transformed into two-dimensional spectrograms using signal processing techniques, allowing the ViT to be directly applied for deep audio representation learning and thus adopted in tasks such as audio deepfake detection. Ulutas et al. [142] employed CQT-generated spectrograms as input and used the ViT for classification, demonstrating that the ViT outperforms CNNs in detecting deepfake audio and surpasses multiple SOTA methods. Similarly, Goel et al. [143] used a self-supervised audio spectrogram Transformer (SSAST) [144,145], a ViT-based architecture, to develop a contrastive learning framework, SSAST-CL, for detecting audio deepfakes. Their approach integrates Siamese networks with self-attention and cross-attention mechanisms to enhance the ViT’s feature representation capabilities. They also employed contrastive loss to distinguish between bona fide and spoofed audio. This approach achieved competitive performance on the ASVspoof 2021 LA dataset, outperforming multiple baseline methods.

Graph neural networks (GNNs) [146] are a class of deep learning models designed specifically to process graph-structured data, effectively capturing complex dependencies between nodes as well as global graph topology. Common GNN variants include Graph Convolutional Networks (GCNs) [147] and Graph Attention Networks (GATs) [148]. Chen et al. [149] pointed out the significant differences in spectro-temporal dependency between genuine and fake audio and proposed leveraging GCNs to model and capture these dependencies. Experimental results demonstrated that the model outperformed all contrast models on the ASVspoof 2019 dataset, validating its effectiveness in addressing the challenges of complex audio deepfake detection tasks. Tak et al. [150] introduced an E2E model, RawGAT-ST, which combines RawNet2 with a GAT to model relationships across different spectral and temporal regions, effectively capturing subtle deepfake artefacts in fake audio. The model achieved SOTA performance on the ASVspoof 2019 dataset, significantly surpassing baseline models, including RawNet2. This study showed the GAT’s powerful ability to model complex spectro-temporal dependencies in spoofed audio and its broad potential in the field of audio deepfake detection. Building on the GAT’s ability to capture spectral and temporal relationships, Jung et al. [151] adopted a heterogeneous stacking graph attention layer (HS-GAL) to integrate information from different domains and learn inter-domain dependencies. Their proposed E2E AASIST model showed improved performance over the previous RawGAT-ST model on the ASVspoof 2019 dataset, demonstrating exceptional generalisation capabilities. In a further advancement, Tak et al. [152] explored the combination of the SSL model W2V2 with GAT to enhance the performance of audio deepfake detection. Experimental results revealed that the AASIST model, combined with W2V2, achieved superior performance on the ASVspoof 2021 LA and DF datasets compared to traditional methods and other baseline models. Notably, the model exhibited excellent generalisation in unseen attack scenarios, highlighting its robustness and practical applicability.

### 5.3. Discussion

Table 5 and Table 6 present a selection of recent audio deepfake detection systems and their performance on the ASVspoof datasets. The systems are ranked based on their EER. By analysing these systems, we can draw key observations into the advancements and trends in this field.

- Data augmentation (DA): Data augmentation expands the dataset by manually altering the original data, thereby diversifying the training set and reducing overfitting to enhance model generalisation. Common audio DA techniques include frequency masking, time-shifting, reverberation, and newly proposed methods such as SpecAug and RawBoost. As observed in the table, many of the high-performing systems in recent years, particularly on the ASVspoof 2021 dataset, have incorporated DA to achieve superior results. Notably, the MelSpec+ABC-CapsNet system proposed by Wani et al. [105] achieved SOTA performance on the ASVspoof 2019 LA dataset without employing any DA. This highlights the possibility that the choice of input features and model architecture may outweigh the impact of augmentation techniques in certain scenarios.
- Frontend and backend selection: The frontend is responsible for feature extraction, forming the foundation of system performance. SSL frontends, such as WavLM and W2V2, have dominated the recent audio deepfake detection landscape. Their ability to capture rich contextual information across both temporal and spectral domains has significantly surpassed traditional handcrafted features like LFCC and CQCC. While handcrafted features remain relevant in resource-constrained scenarios due to their simplicity and efficiency, the prevailing trend clearly favours SSL features for their superior adaptability to unseen attacks. The backend classifier determines how effectively the extracted features are utilised for classification. Traditional machine learning models, such as the GMM and SVM, are no longer the focus of contemporary research due to their limited ability to handle complex spoofing attacks. In contrast, advanced deep learning models, such as CapsNet and GNN, as well as hybrid architectures, have shown remarkable capabilities in capturing intricate temporal and spectral dependencies. Studies by Wani et al. [105] and Ulutas et al. [142] have further demonstrated that combining SOTA backend models with traditional handcrafted features can outperform systems utilising learning-based features alone, underscoring the importance of a well-chosen backend in achieving optimal performance.
- Generalisation: Table 7 presents the generalisation ability of various models on the ITW dataset. It emphasises the pivotal role of DA in not only improving model performance but also enhancing cross-domain robustness. For instance, models employing DA, such as XLS-R and W2V2+MoE Fusion, achieved significantly lower EERs on out-of-domain datasets compared to those without DA. This underscores that well-designed DA strategies can effectively enhance a model’s resilience against unseen attacks. Furthermore, SSL feature extraction methods, with their ability to comprehensively capture temporal and spectral information, exhibited superior adaptability and robustness compared to traditional handcrafted features. This proves the advantage of SSL features in addressing complex deepfake attacks and stresses their critical role in improving model robustness to unseen scenarios.

**Table 5:** Comparison of model performances on ASVspoof 2019 LA dataset (sorted by EER). DA stands for data augmentation. ✓ indicates that data augmentation was applied.

Year	DA	Frontend	Backend	EER (%)	t-DCF
2024 [105]		MelSpec+VGG18	ABC-CapsNet	0.06	
2023 [142]		CQT	ViT	0.19	0.1102
2024 [131]	✓	SLIM Framework		0.2	
2022 [153]		W2V2	VIB	0.4	0.0107
2024 [129]		WavLM	MFA	0.42	0.0126
2023 [149]	✓	LFB	GCN	0.71	0.0192
2024 [154]	✓	W2V2	MoE Fusion	0.74	
2024 [127]		W2V2	SVM	0.9	
2022 [155]		W2V2+Light-DARTS		1.08	
2024 [127]		W2V2	MLP	1.11	
2022 [151]		AASIST		1.13	0.0347
2024 [156]		RawBMamba		1.19	0.036
2024 [127]		W2V2	LR	1.25	
2021 [150]	✓	RawGAT-ST		1.39	0.0443
2024 [156]		RawMamba		1.47	0.0467
2021 [108]		Spec	Attn-CNN-OC-Softmax	1.87	0.051
2024 [115]		LFCC+MPE	SENet	1.94	
2021 [114]		LFCC	CapsNet	1.97	0.0538
2021 [113]		LFCC	ResNet18-OC-Softmax	2.19	0.059
2024 [115]		LFCC+MPE	LCNN	2.41	
2022 [122]		RawNet2	STATNet	2.45	0.062
2024 [130]		WavLM+MTB	MLP	2.47	
2021 [114]		LPS	CapsNet	3.19	0.0982
2021 [110]	✓	LPS	TE-ResNet	6.02	
2021 [110]	✓	MFCC	TE-ResNet	6.54	
2021 [110]	✓	CQCC	TE-ResNet	7.14	
2024 [130]		W2V2+MTB	MLP	16.4	

**Table 6:** Comparison of model performances on ASVspoof 2021 LA and DF datasets (sorted by DF EER). DA stands for data augmentation. ✓ indicates that data augmentation was applied.

Year	DA	Frontend	Backend	LA (EER %)	LA (t-DCF)	DF (EER %)
2024 [117]	✓	XLS-R	SLS	3.88		2.09
2024 [154]	✓	W2V2	MoE Fusion	2.96		2.54
2024 [129]		WavLM	MFA	5.08		2.56
2022 [152]	✓	W2V2	SA	1	0.2066	3.69
2024 [131]	✓	SLIM Framework				4.4
2022 [126]	✓	W2V2	MLP+ASP	3.54		4.98
2022 [155]		W2V2+Light-DARTS				7.86
2024 [130]		WavLM+MTB	MLP			9.87
2024 [156]		RawBMamba		3.28	0.2709	15.85
2023 [119]	✓	MelSpec+SincNet	Transformer-Bi-Level			20.24
2024 [156]		RawMamba		2.84	0.2517	22.48
2024 [130]		W2V2+MTB	MLP			27.1

**Table 7:** Comparison of model performances on In-the-Wild dataset (sorted by EER). DA stands for data augmentation. T and D stand for training and development partitions, respectively. ✓ indicates that data augmentation was applied.

Year	DA	Frontend	Backend	Training Data	EER (%)
2024 [117]	✓	XLS-R	SLS	ASVspoof 2019 LA (T)	8.87
2024 [154]	✓	W2V2	MoE Fusion	ASVspoof 2019 LA (T)	9.17
2024 [131]	✓	SLIM Framework		S1: Common Voice, RAVDESS S2: ASVspoof 2019 LA (T)	12.5
2024 [116]		Fusion	ResNet18	ASVspoof 2019 LA (T, D)	24.27
2023 [107]	✓	C-CQT	CVNN	ASVspoof 2019 LA (all splits)	26.95 ± 3.12
2024 [116]		Hubert	ResNet18	ASVspoof 2019 LA (T, D)	27.48
2024 [115]		MPE	SENet	ASVspoof 2019 LA (T)	29.62

## 6. Emerging Research Directions

Beyond improving the performance of audio deepfake detection models, recent research has increasingly focused on critical aspects like privacy, fairness, continual learning, and explainability. These emerging directions emphasise the importance of addressing broader challenges in the field beyond detection accuracy.

### 6.1. Privacy

Considering the increasing importance of data security and privacy [157], Li et al. [90] proposed the first content-privacy-preserving audio deepfake detection framework called SafeEar. SafeEar uses a HuBERT-equipped multilayer Residual Vector Quantisers (RVQs) to decouple semantic and acoustic features within speech signals, using only the acoustic features as input to the detection model. To further enhance the concealment of semantic information, the framework applies random shuffling of the temporal order of acoustic information and dimensionality reduction of acoustic tokens, preventing both humans and machines from reconstructing the original content. This design enhances resilience against content recovery attacks while maintaining competitive detection performance. SafeEar achieves comparable accuracy to mainstream methods on the ASVspoof 2019 LA dataset and outperforms most baselines on the ASVspoof 2021 LA dataset. Moreover, in adversarial scenarios where attackers have access to automatic speech recognition (ASR) models, knowledge of SafeEar’s algorithm, or even its shuffling order, SafeEar forces ASR models to exceed 90% word error rate, demonstrating its robustness in preventing unauthorised content recovery. However, SafeEar has certain limitations. Its Transformer-based detection model incurs high computational costs, making it less suitable for resource-constrained environments. It also exhibits reduced generalisation in cross-language scenarios and decreased robustness in unknown codec conditions. Despite these challenges, SafeEar introduces a novel acoustic-only approach for privacy-preserving deepfake detection, offering a foundation for future research in secure and explainable detection techniques.

### 6.2. Fairness

Yadav et al. [158] discussed the fairness problem in synthetic speech detection (SSD) systems, focusing on biases against various demographic groups. The study evaluated six SSD systems, covering handcrafted feature-based to learning-based and E2E approaches. They constructed an evaluation dataset by processing bona fide speech samples from Mozilla Common Voice Corpora (CVC) [159] and Sep-28K [160], covering diverse genders, ages, and accents. The results revealed that SSD systems are biased, exhibiting higher false-positive rates for bona fide speech from male speakers, adolescents, and the elderly, as well as speakers with South Asian and Australian English accents. Additionally, SSD systems were found to be particularly unfair to speech-impaired speakers. The study underscores the need for the multimedia forensics community to address these biases and develop more equitable synthetic speech detection systems to mitigate potential unfairness to specific demographic groups.

### 6.3. Adaptability

With the rapid development of speech synthesis technologies, existing audio deepfake detection systems often experience performance degradation when faced with new spoofing attacks. To address this challenge, continual learning frameworks have been widely adopted, aiming to enable effective detection of unseen spoofing attacks while retaining knowledge of previously learned attack types. Zhang et al. [161] observed that the feature distribution of real speech across different tasks is relatively compact, whereas the distribution of fake speech features is more dispersed. Based on this observation, they utilised in-class cosine distance to quantify the feature distribution and proposed a continual learning method based on Radian Weight Modification (RWM). By dynamically adjusting gradient directions, RWM effectively balances the acquisition of new knowledge and the preservation of prior knowledge. Experimental results demonstrated that RWM significantly outperforms mainstream continual learning methods in terms of knowledge memorisation. Moreover, RWM exhibited strong adaptability and robustness in image recognition tasks, showcasing its potential for broader applications. Dong et al. [162] introduced the Continual Audio Defense Enhancer (CADE) framework, which incorporates a fixed memory size to store a subset of previously encountered data, ensuring the preservation of knowledge when new attack types are introduced. The framework also integrates three innovative loss functions: Knowledge Distillation Loss to align predictions between the old and new models, Attention Distillation Loss to capture critical feature regions, and Positive Sample Alignment Loss to enhance the generalisation ability of the model to genuine audio. Experimental results revealed that CADE not only efficiently detects new types of spoofed audio but also prevents the forgetting of previously learned deepfake features, making it particularly suitable for real-world scenarios with continuously evolving spoofing types. Furthermore, Zhang et al. [163] proposed the EVolving synthetic and Deepfake Audio detection (EVDA) benchmark, designed specifically to evaluate continual learning methods for audio deepfake detection. The EVDA benchmark combines classical datasets with novel deepfake audio data to create a comprehensive and challenging testing environment. The study’s core contribution lies in establishing a standardised platform that facilitates the testing and development of continual learning models capable of addressing the challenges of audio deepfake detection while adapting to emerging generative technologies.

### 6.4. Explainability

The inherent “black-box” nature of neural networks presents significant challenges in terms of user trust, model optimisation, and ethical compliance. In audio deepfake detection and other AI tasks, explainability not only is crucial for enhancing system transparency and reliability but also serves as a powerful tool for researchers to identify model weaknesses, optimise feature selection, and refine model design. SHapley Additive ExPlanations (SHAP) [164] is a unified framework for explaining model predictions. By quantifying the contribution of each feature to a specific prediction, SHAP provides insights into model behaviour. Ge et al. [165,166] pioneered the use of SHAP in the domain of audio deepfake detection to compare the behaviours of two detection models. Their findings revealed significant differences in how these models focused on speech and nonspeech intervals, with some models relying more heavily on silence duration rather than speech content to determine authenticity [167]. Furthermore, the study demonstrated that different types of spoofing attacks often leave unique traces in specific frequency bands, suggesting that detection models can significantly enhance their discrimination capabilities by concentrating on these regions. Channing et al. [168] introduced an attention roll-out mechanism for Transformer-based audio deepfake detectors. By accumulating attention weights across multiple layers, this method visualises the distribution of model attention, enabling researchers to intuitively observe audio segments the model focuses on during decision-making. This approach improves model transparency and meanwhile offers critical insights into the behaviour of Transformer models in complex tasks, thereby improving both reliability and applicability.

### 6.5. Anti-Spoofing

Yu et al. [169] proposed AntiFake, a proactive defence mechanism that embeds imperceptible adversarial perturbations into targets’ audio samples to disrupt attackers’ speech synthesis models. These perturbations have negligible impact on human auditory perception but cause the synthesised audio to mimic a different speaker’s voice rather than that of the target. Experimental results demonstrate that AntiFake achieves over a 95% protection rate across five contemporary speech synthesisers and three speaker verification systems. Furthermore, AntiFake remains robust against adaptive attacks, including speech sample transformations and optimisation-based perturbation removal, providing an innovative and effective countermeasure against unauthorised speech synthesis. Juvela and Wang [170] introduced a collaborative training-based audio watermarking technique, which enables the generation model to embed watermarks actively, making the synthesised speech more easily identifiable by the collaboratively trained detection model while maintaining high audio quality and naturalness. Experimental results demonstrate that compared to traditional digital signal processing-based watermarking, the detection model trained with this scheme exhibits superior robustness against adversarial conditions, such as time-stretching and noise addition. Furthermore, the subjective mean opinion score (MOS) indicates that the collaborative training process has minimal impact on the naturalness of the audio, rendering the watermarked samples almost indistinguishable from the original ones. Liu et al. [171] further proposed a generalised Timbre Watermarking technique to enhance the detection of diverse voice-cloning attacks. This approach embeds watermarks in the frequency domain, leveraging its inherent robustness to audio processing operations. Additionally, the study incorporates a distortion layer that simulates common processing operations in voice-cloning attacks, which, when integrated into the E2E training process, improves the watermark’s robustness. Experimental results show that the watermark extraction accuracy remains at 100% against preprocessing attacks such as resampling and compression while maintaining nearly 90% accuracy under extreme conditions like low-bitrate compression and low-pass filtering. Even when only 75% of the training data are watermarked, cloned voices can still be effectively detected. Furthermore, the method exhibits strong resistance to advanced adversarial attacks targeting watermark integrity, such as watermark overwriting and removal attacks. Importantly, the watermarked audio maintains a quality nearly identical to the original, demonstrating an excellent balance between fidelity and anti-spoofing capability.

### 6.6. Robustness Against Compression

Unlike most audio deepfake detection systems that rely on high-quality training datasets, such as ASVspoof 2019, which uses FLAC for lossless compression [72], real-world applications (e.g., social media and voice communication) typically involve audio that has undergone lossy compression, such as MP3, AAC, and Opus. While these lossy compression techniques significantly reduce storage and transmission costs, they also lead to the loss of certain frequency- and time-domain information [172], thereby reducing the robustness of audio deepfake detection systems, particularly in real-time detection scenarios. To address this challenge, Li et al. [90] integrated multiple representative audio codecs into the training pipeline to mitigate the disruptive effects of codecs. Furthermore, Wang et al. [172] introduced Frequency–Time Domain Knowledge Distillation (FTDKD), which utilises knowledge distillation to enable student models trained on low-quality audio to effectively learn the characteristics of high-quality audio more effectively. Their experiments covered ten different codecs and demonstrated that FTDKD achieved excellent performance on both the ASVspoof 2019 LA and 2021 DF datasets, showcasing its superior generalisation capability across both high- and low-quality audio environments. Xiang et al. [173,174] further proposed Efficient Spectrograms (E-Specs), a novel feature extraction method specifically designed for MP3-compressed speech. This approach enables the direct extraction of spectral features from the MP3 compression domain without full decoding, leading to reduced decoding time and significantly lower computational complexity. Experimental results demonstrate that when using the same backend model, E-Specs outperform other handcrafted features.

### 6.7. Real-World Implications

The rise of deepfake technology poses severe threats to security and trust, particularly in financial and cybersecurity systems. In 2024, cybercriminals attempted to impersonate the CEO of WPP [175], using deepfake audio to authorise fraudulent transactions, exposing the vulnerabilities of voice authentication. To combat such threats, companies like Reality Defender [176] have developed real-time deepfake detection specifically designed for integration into financial security frameworks. By deploying AI-powered audio analysis, these systems can detect synthetic speech and identify anomalies in real time, providing an additional security layer for institutions that rely on voice-based authentication. Financial institutions have started integrating such solutions into their customer service and fraud prevention workflows, ensuring that attackers cannot bypass biometric authentication through voice cloning.

Beyond financial fraud, deepfake manipulation extends to multimedia manipulation, prompting the need for multimodal deepfake detection solutions. Resemble AI [177], for instance, has introduced a detection platform capable of analysing audio, images, and videos simultaneously, reinforcing media authentication and misinformation prevention. This ability to process deepfake content in real time makes the solutions particularly relevant in high-stakes environments where immediate verification is critical, such as political campaigns and corporate security. At the consumer level, McAfee launched “Deepfake Detector”, an AI tool designed to detect deepfake audio in real time [178]. This software scans audio content in videos and alerts users when synthetic speech is detected, achieving an impressive 96% accuracy rate. While currently focused on audio content, its development signifies a broader industry effort to equip individuals with accessible deepfake detection capabilities.

These real-world applications demonstrate that audio deepfake detection is no longer just an academic challenge but a critical technological necessity. The increasing adoption of detection systems in financial institutions, cybersecurity frameworks, and consumer-level applications highlights the evolving nature of deepfake threats and the corresponding need for continuous advancements in detection methodologies. As deepfake generation technologies become more sophisticated, detection systems must adapt to remain effective, incorporating real-time processing, cross-modal analysis, and robust AI techniques to safeguard digital security.

## 7. Future Research

With the rapid development of deep learning technologies, audio deepfake generation has become increasingly sophisticated, posing major challenges to both societal and technical domains. As a crucial area of research, audio deepfake detection has made notable progress in recent years. However, several key limitations remain, and future research can focus on the following directions to address these challenges:

Enhancing generalisation and adaptability: Although current detection models perform well on in-domain data, their ability to generalise across languages, domains, or novel spoofing attacks remains limited, restricting their deployment in real-world applications. Future efforts could solve this by developing more targeted augmentation techniques or leveraging unsupervised or weakly supervised transfer learning approaches. Furthermore, integrating the complementary strengths of humans and machines [27] could enhance detection systems, with models focusing on areas where human judgement struggles, thereby improving adaptability and robustness.

Education and training: Given the prolific usage of deepfakes to perpetrate scams and spread misinformation, it is essential to educate the general public on how to recognise and respond to the usage of these fakes [179]. Effective training programs and awareness campaigns should be developed to improve individuals’ ability to identify deepfake content and understand its potential risks. This is critical as deepfakes have the power to trick and persuade public opinion in harmful ways. Collaboration among educators, policymakers, and technologists can ensure that these initiatives are practical and tailored to diverse audiences.

Developing lightweight models: High-performing detection systems often depend on large-scale deep learning architectures, making them unsuitable for resource-constrained settings, such as mobile devices or embedded systems. While a study by Saha et al. [127] reduced model parameters by integrating pretrained SSL models with traditional machine learning techniques, the storage demands of large SSL models remain a bottleneck. Future work can address this through model pruning and quantisation to reduce storage and computational overhead or creating compact and computationally efficient model architectures suitable for real-time detection.

Improving dataset diversity and fairness: Although existing datasets are expanding in size, they still lack sufficient coverage of real-world scenarios, such as diverse languages, accents, background noises, and device conditions. Furthermore, imbalanced data distributions in terms of speaker types can lead to performance biases, raising concerns about fairness. Future work could focus on adopting voice conversion techniques to introduce diverse accents and utilising crowdsourcing to expand speaker variation. Additionally, existing datasets can be enhanced by incorporating data augmentation techniques, such as adding background noise or applying transmission channel distortions. To further improve model robustness and fairness, it is also important to integrate fairness evaluation frameworks to identify performance disparities across speaker groups.

Ethical frameworks and policies: The rapid advancement of AI and deepfake technologies has outpaced the establishment of corresponding legal and regulatory frameworks, creating potential ethical and societal risks. Future research should focus on establishing comprehensive ethical guidelines and standards to promote the responsible use of deepfake technology. This includes defining acceptable use cases, enhancing transparency and accountability between developers and users, and promoting collaboration among technologists, policymakers, and ethicists to ensure that these technologies are applied in ways that benefit society.

Improving Model explainability: Many high-performance detection models still operate as “black boxes”, offering limited transparency into their decision-making processes. This lack of interpretability hinders user trust and raises ethical and legal concerns. While some studies have explored explainability in audio deepfake detection, these methods are often unified frameworks or tailored to specific models, making them less effective in revealing task-specific decision principles. Future research should aim to develop explainability frameworks tailored to audio deepfake detection in real time, focusing on key features such as anomalies in frequency bands or temporal characteristics that indicate deepfakes. Furthermore, future research could explore multiview feature fusion and scenario-based dynamic feature selection strategies to better integrate the interpretability of handcrafted features with the robustness of learning-based features, thereby enhancing the trustworthiness of audio deepfake detection systems.

## 8. Conclusions

This survey provides a comprehensive overview of the latest techniques, datasets, and challenges in audio deepfake detection. It examines various detection methodologies, from feature extraction to model design, highlighting their comparative strengths and limitations. Additionally, it explores advanced topics such as privacy, fairness, explainability, and adaptability, emphasising the multidimensional nature of this research area. Despite impressive progress, critical gaps remain, particularly in improving generalisation to unseen attacks, developing lightweight models for resource-constrained environments, and ensuring dataset diversity to better reflect real-world conditions. Addressing these challenges requires adaptable, efficient, and ethically responsible solutions that meet the demands of practical deployment. By summarising existing knowledge and identifying open problems, this survey aims to serve as a valuable resource for researchers and practitioners, guiding future innovation in the rapidly evolving field of audio deepfake detection.

## Figures and Tables

**Table 1:** Comparison of existing audio deepfake detection surveys and our contributions.

Authors	Year	Main Contributions	Limitations
Chadha et al. [4]	2021	Introduces deepfake technology, types, and detection methods, focusing mainly on image and video deepfakes.	Limited focus on audio deepfake detection, lacks evaluation of modern detection models and benchmark comparisons.
Ren et al. [36]	2021	Discusses deepfake attacks on both human perception and ASV systems.	Lacks analysis of advanced detection models, limited discussion on explainability or privacy in deepfake detection.
Almutairi and Elgibreen [33]	2022	Categorises ML/DL techniques, analyses datasets, and discusses future challenges.	Limited exploration of advanced detection models.
Khanjani et al. [32]	2023	Categorises generation and detection methods, reviews over 150 studies, and discusses societal threats.	Lacks a quantitative comparison of detection models, does not cover recent advancements beyond 2021.
Patel et al. [34]	2023	Provides a multimodal deepfake analysis, introduces a case study on detection inconsistencies.	Lacks a detailed technical evaluation of audio deepfake detection methods, does not provide quantitative model benchmarking.
Yi et al. [35]	2023	Performs an experimental comparison on benchmark datasets.	Lacks real-world application discussions and in-depth analysis of robustness and explainability.
Mubarak et al. [37]	2023	Emphasises societal impacts and holistic mitigation strategies, discusses detection techniques and emerging threats.	Takes a generalised approach across media types, does not include recent technological advances in audio detection models.
Masood et al. [38]	2023	Provides a detailed review of deepfakes across modalities, discusses datasets, detection methods, and future challenges.	Lacks in-depth technical analysis specific to audio deepfake detection, does not benchmark detection models across datasets.
Ours	2024	Most up-to-date survey (till 2024); provides a quantitative comparison of detection models; first to analyse privacy, fairness, adaptability, and explainability in audio deepfake detection. Expands beyond detection methods to robustness, real-world deployment, and future research directions.	

**Table 2:** Comparison of speech synthesis and voice conversion techniques.

Attribute	Speech Synthesis	Voice Conversion
Objective	Generate speech from nonspeech input	Transform input speech into target speech
Input	Text, phonemes, etc.	Source speech signal
Output	Speech signal	Speech signal with consistent content but altered style or attributes
Complexity	Language understanding and speech generation	Feature transformation and target reconstruction
Representative models	DeepVoice, Tacotron, FastSpeech	CycleGAN-VC, AutoVC, MulliVC, VQVC, FreeVC
Main challenges	Enhancing speech naturalness and fluency	Maintaining content consistency while changing target attributes

**Table 3:** Baseline performance of ASVspoof and ADD challenges ^1^.

Challenge	Language	Year	Frontend	Backend	Performance		
ASVspoof	English	2019			tDCF		EER(%)
			CQCC	GMM	0.2839		9.57
			LFCC	GMM	0.2605		8.09
		2021			LA		DF
					tDCF	EER(%)	EER(%)
			CQCC	GMM	0.4974	15.62	25.56
			LFCC	GMM	0.5758	19.3	25.25
			LFCC	LCNN	0.3445	9.26	23.48
			RawNet2		0.4257	9.5	22.38
		2024			CM		SASV
					minDCF	EER(%)	a-DCF
			RawNet2		0.8266	36.04	
			AASIST		0.7106	29.12	
			Fusion-based				0.6806
			Single integrated				0.5741
ADD	Chinese	2022 ^2^			LF (EER %)		PF (EER %)
			LFCC	GMM	25.2		45.8
			LFCC	GMM	24.1		47.5
			LFCC	LCNN	32.3		47.8
			LFCC	LCNN	29.9		48.1
			RawNet2		35.2		50.1
			RawNet2		33.9		50.2
		2023			FG-D (WEER ^3^ %)		
			LFCC	GMM	53.04		
			LFCC	LCNN	66.72		
			Wav2Vec2	LCNN	30.05		

^1^ ASVspoof 2015 and 2017 are excluded for the following reasons: (1) both have outdated datasets and limited relevance to current research trends; and (2) ASVspoof 2017 solely focuses on replay attacks, which is beyond the scope of this paper. ^2^ The upper rows of the same settings use only training data, while the lower rows incorporate adaption data. ^3^ Weighted EER, reflecting the weighted results across two rounds of FG-D evaluation.

## Data Availability

No new data were created or analyzed in this study. Data sharing is not applicable to this article.
